# Epidemiological profile, completeness of notifications, and factors associated with the adequacy of treatment for syphilis during pregnancy: a cross-sectional study, Ubá, 2018–2023

**DOI:** 10.1590/S2237-96222026v35e20250056.en

**Published:** 2026-03-20

**Authors:** Priscila Teixeira Silva, Luana Vieira Toledo, Brunnella Alcantara Chagas de Freitas, Valker Araújo Feitosa

**Affiliations:** 1 Universidade Federal de Viçosa, Departamento de Medicina e Enfermagem, Viçosa, MG, Brazil; 2 Universidade de São Paulo, Faculdade de Ciências Farmacêuticas, São Paulo, SP, Brazil

**Keywords:** Syphilis, Pregnant People, Sexually Transmitted Diseases, Disease Notification, Cross-Sectional Studies, Sífilis, Personas Embarazadas, Enfermedades de Transmisión Sexual, Notificación de Enfermedades, Estudios Transversales

## Abstract

**Objective:**

To analyze the epidemiological profile of syphilis during pregnancy, the completeness of notifications, and the factors associated with treatment adequacy in Ubá, a region of Minas Gerais, between 2018 and 2023.

**Methods:**

Cross-sectional study with records from the Brazilian Notifiable Diseases Information System (SINAN). The annual detection rate of syphilis during pregnancy and logistic regression to assess treatment were calculated. Data completeness was calculated and categorized.

**Results:**

The detection rate ranged from 17.7 cases per 1 thousand live births in 2019 to 41.3 in 2022. Pregnant women had a median age of 23 years (interquartile range 19; 28), with 61.9% identifying as Black and 36.1% diagnosed in the third trimester. Penicillin was indicated in 75.3% of cases, and partner treatment occurred in 41.2%. Completeness was excellent for the variables “pregnancy trimester at diagnosis” (100.0%) and “nontreponemal test” (98.9%); poor for “education level” (65.4%) and “clinical classification” (70.6%). Inadequate prescriptions were higher among pregnant people diagnosed in the third trimester than among those diagnosed in the first trimester (*odds ratio* [OR] 2.00; 95% confidence interval [95%CI].30; 3.07). Pregnant people with a nonreactive result for the nontreponemal test (OR 13.48; 95%CI 5.93; 30.64) and those who did not undergo the treponemal test (OR 1.65; 95%CI 1.13; 2.40) had higher odds of inadequate prescription. There were more prescription failures among pregnant people whose sexual partners were not treated (OR 1.91; 95%CI 1.34; 2.72).

**Conclusion:**

A high detection rate and failures in notification completeness and in the treatment prescribed for pregnant people with syphilis were observed.

## Resumen

**Objetivo:**

Analizar el perfil epidemiológico de la sífilis gestacional, la completitud de las notificaciones y los factores asociados a la adecuación del tratamiento en Ubá, región de Minas Gerais, entre 2018 e 2023.

**Métodos:**

Estudio transversal con registros del Sistema de Información de Enfermedades de Notificación (SINAN). Se calculó la tasa anual de detección de sífilis durante el embarazo y se empleó regresión logística para evaluar el tratamiento. La completitud de los datos fue calculada y categorizada.

**Resultados:**

La tasa de detección osciló entre 17,7 casos por 1.000 nacimientos vivos en 2019 y 41,3 en 2022. Las personas embarazadas presentaron una mediana de 23 años (rango intercuartílico 19; 28), siendo el 61,9% de raza/color de piel negra y el 36,1% diagnosticadas en el tercer trimestre. La penicilina fue indicada en el 75,3% de los casos, y el tratamiento de la pareja ocurrió en el 41,2%. La completitud fue excelente para las variables “período gestacional del diagnóstico” (100,0%) y “prueba no treponémica” (98,9%); y baja para “nivel educativo” (65,4%) y “clasificación clínica” (70,6%). La prescripción inadecuada fue mayor entre personas embarazadas diagnosticadas en el tercer trimestre en comparación con el primer trimestre (*odds ratio* [OR] 2,00; intervalo de confianza del 95% [IC95%] 1,30; 3,07). Las personas embarazadas con resultado no reactivo en la prueba no treponémica (OR 13,48; IC95% 5,93; 30,64) y aquellas que no realizaron la prueba treponémica (OR 1,65; IC95% 1,13; 2,40) presentaron mayores probabilidades de prescripción inadecuada. Se observaron más fallas de prescripción entre gestantes cuyas parejas sexuales no fueron tratadas (OR 1,91; IC95% 1,34; 2,72).

**Conclusión:**

Se observaron una elevada tasa de detección y fallas tanto en la completitud de las notificaciones como en el tratamiento prescrito a las personas embarazadas con sífilis.

**Ethical aspects**

This research respected ethical principles, having obtained the following approval data:

Research ethics committee: Universidade Federal de Viçosa

Opinion number: 6.312.070

Approval date: 20/9/2023

Certificate of submission for ethical appraisal: 73354323.9.0000.5153

Informed consent form: Waived.

## Introduction 

Syphilis is a centuries-old disease that persists as a public health challenge. One of the main concerns for controlling this sexually transmitted infection is its occurrence in pregnant people, who can transmit it to the fetus, resulting in congenital syphilis. Vertical transmission is preventable through early diagnosis and adequate treatment of pregnant people during prenatal care (1).

Despite extensive knowledge about preventive strategies, the availability of sensitive tests, and effective low-cost treatment, syphilis is the second leading cause of preventable stillbirths worldwide. The global estimate in 2022 was 700 thousand cases of congenital syphilis and more than 200 thousand fetal and neonatal deaths (2).

In 2007, the World Health Organization (WHO) launched an initiative to eliminate vertical transmission to increase access to testing and treatment for pregnant people. An official validation process was established so that countries could be recognized upon reaching this goal, and an incidence of up to 0.5 cases of congenital syphilis per 1 thousand live births was considered acceptable (3). 

Brazil has faced numbers far from the elimination targets. In 2023, it recorded an incidence of 34 cases of syphilis during pregnancy and approximately 10 cases of congenital syphilis per 1 thousand live births (4).

National data on syphilis come from the Brazilian Notifiable Diseases Information System (*Sistema de Informação de Agravos de Notificação*, SINAN). Compulsory notification occurs through the completion of standardized forms by the health professional who identifies the case, followed by submission to the epidemiological surveillance service for data entry into SINAN. 

Syphilis surveillance is an important monitoring tool that enables tracking the disease’s behavior and supports the planning of prevention and control measures (5). The quality of the records, as reflected in the completeness and consistency of all reported data, is essential for health indicators to fulfill their purposes (6).

Minas Gerais has reported a gradual increase in syphilis cases, and the inadequate treatment of pregnant people has proven to be an important challenge to be overcome. There is a notable fragility in the completeness of notifications and their implications for planning priority actions, highlighting the need to improve the database (7). In 2023, the state recorded more than 30 cases of syphilis during pregnancy and approximately 10 cases of congenital syphilis per 1 thousand live births (7). Since 2021, the Regional Health Management Department of Ubá has shown rates higher than state and national levels (4,7,8).

Characterizing cases and identifying factors related to the quality of care in a region is essential for formulating effective health policies (9). Diagnosing the regional situation highlights the real needs of the population in question and allows the proposal of actions that consider its particularities in order to advance syphilis prevention and control.

This study aimed to analyze the epidemiological profile of syphilis during pregnancy, the completeness of notifications, and the factors associated with treatment adequacy in Ubá, Minas Gerais, between 2018 and 2023.

## Methods 

### Study design

This is a cross-sectional, descriptive-analytical study based on notifications of syphilis during pregnancy recorded in SINAN, among residents assigned to the Regional Health Management Department of Ubá, Minas Gerais.

### Setting 

The 583 municipalities of Minas Gerais are organized into 14 health macroregions and 89 health microregions to structure service provision within their respective areas of coverage. 

The Regional Health Management Department of Ubá is located in the Zona da Mata and is part of the Southeast macroregion. This regional health department encompasses: (i) the Muriaé microregion, composed of 11 municipalities, with an estimated population of 173,744 inhabitants in 2020; and (ii) the Ubá microregion, comprising 20 municipalities with an estimated population of 314,647 inhabitants in 2020 (10). 

Its definition as the setting of this study was justified by the epidemiological relevance of the territory, which has been marked by high incidences of syphilis, surpassing state and national rates (4,7,8).

### Participants 

The study included cases of syphilis during pregnancy notified among residents of the 31 municipalities that make up the Regional Health Management Department, with diagnoses between 2018 and 2023. The study period was defined to standardize the case definition criteria, which were updated in 2017. A case of syphilis during pregnancy was defined as a woman who, during prenatal care, childbirth, or the postpartum period, presented reactive treponemal and nontreponemal test(s), regardless of symptoms (11).

Notifications automatically classified by the system as “excluded” for not meeting definition criteria were excluded.

### Variables 

Notification of syphilis during pregnancy was completed by health professionals using a standardized form. The study considered the following variables from the form: age; race/skin color (White, Black, Asian, Brown [Brazilian mixed race], Indigenous); education level (illiterate; incomplete 1st–4th grades of elementary school; completed 4th grade of elementary school; incomplete 5th–8th grade of elementary school; complete elementary school; incomplete high school; complete high school; incomplete higher education; complete higher education); pregnancy trimester at diagnosis (first trimester, second trimester, third trimester, gestational age unknown); clinical classification of syphilis (primary, secondary, tertiary, latent); nontreponemal test (reactive, nonreactive, not performed); treponemal test (reactive, nonreactive, not performed); treatment prescribed to the pregnant person (2.4 million international units of benzathine penicillin, 4.8 million international units of benzathine penicillin, 7.2 million international units of benzathine penicillin, another regimen, not performed); and sexual partner treated (yes, no).

These variables were selected because completion is mandatory or essential, and they are fundamental for case surveillance and for calculating epidemiological and operational indicators.

Based on the variables “clinical classification of syphilis” and “treatment prescribed to the pregnant woman,” the researchers assessed whether the prescription was adequate for the clinical stage of infection, considering the Clinical Protocols and Therapeutic Guidelines (*Protocolos Clínicos e Diretrizes Terapêuticas*, PCDT) of the Ministry of Health (MS). The assessment was carried out manually and double-checked. The following were considered adequate: (i) one, two, or three doses of 2.4 million international units of benzathine penicillin in cases of primary and secondary syphilis; and (ii) three doses of 2.4 million international units of benzathine penicillin in cases of latent, tertiary syphilis, and when the variable “clinical classification of syphilis” was marked as unknown/blank (12). Based on this classification, the variable “adequate treatment prescription” was created, with the categories “yes” and “no.” 

In addition to inadequate prescriptions, the “no” category also included cases in which the field “treatment prescribed to the pregnant person” was marked as “not performed.” This decision followed the indicator adopted by the MS to monitor coverage of pregnant people adequately treated for syphilis, which considered both situations as inadequate because they do not prevent vertical transmission (12).

### Data source and measurement

Notification data were collected from the nominal SINAN database of the Regional Health Management Department on 1 March 2024. Data on live births were extracted on 31 October 2024, from the database made publicly available by the Brazilian Unified Health System Information Technology Department (*Departamento de Informática do Sistema Único de Saúde*, DATASUS), accessible through the Health Surveillance Portal of Minas Gerais (13), applying filters for year of birth and the microregion of interest.

Some adjustments were made to the database extracted from SINAN to improve the analysis and evaluation of the results.

- Duplicate records were identified and excluded when a case had been notified more than once during the same pregnancy. For the identification of duplicates, the following were considered: name, mother’s name, date of birth, and date of notification. In cases of duplicate records, only the first record entered into the database was retained.
- The categories “Black” and “Brown” were grouped as “Black” in the race/skin color variable, according to the classification used by the Brazilian Institute of Geography and Statistics (*Instituto Brasileiro de Geografia e Estatística*, IBGE).
- The “education level” variable was grouped into: illiterate, incomplete elementary education, complete elementary education, complete high school, and complete higher education.
- The “treatment prescribed to the pregnant person” variable was grouped into: benzathine penicillin, other treatment regimen, and not performed.

### Statistical methods

The annual detection rate of syphilis in pregnant people was calculated using the absolute number of cases as the numerator and the number of live births as the denominator, multiplied by 1,000.

The variables of interest for characterizing the clinical-epidemiological profile were described using absolute and relative frequencies, median, and interquartile range. In the descriptive analysis, “missing data” were included in the “unknown” category. To assess completeness, the percentage of completion for each variable was calculated. Fields with missing information and fields filled with the category “unknown” were considered incomplete. Completeness was categorized according to the following classification: excellent (completeness>95%), good (completeness 90%–95%), fair (completeness 80%–90%), poor (completeness 50%–80%), and very poor (completeness<50%) (14).

The dependent variable was “adequate treatment prescription.” In the bivariate analysis, the association of each independent variable was assessed using Pearson’s chi-square test. When contingency tables had expected frequencies lower than five, Fisher’s exact test was used. Missing and unknown data for independent variables were excluded from this stage. 

In the multivariate analysis, logistic regression was used to estimate the magnitude of the association between the independent variables and the adequate treatment prescription (dependent variable). The *odds ratio* (OR) and 95% confidence intervals (95%CI) were calculated. Model fit was assessed using the Hosmer–Lemeshow test (p-value 0.657). Statistical significance was evaluated using Wald’s test, with p-value<0.05. All analyses were performed using IBM SPSS Statistics.

## Results 

**Figure 1:**
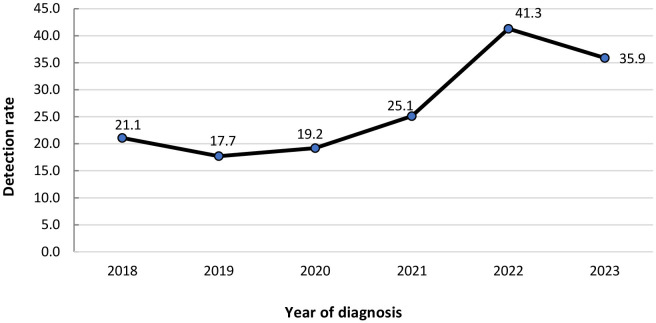
Detection rate of syphilis during pregnancy (cases per 1 thousand live births), by year of diagnosis. Regional Health Management Department of Ubá, 2018–2023 (n=837)

The original database contained 853 records. Four cases classified as “excluded” and 12 duplicate records were removed from the analysis. Between 2018 and 2023, 837 cases of syphilis during pregnancy were identified, with the highest detection rate observed in 2022, 41.3 cases per 1 thousand live births (Figure 1).

Pregnant people with syphilis had a median age of 23 years (interquartile range 19–28), and 61.9% were Black. It was observed that 36.1% of diagnoses occurred in the third pregnancy trimester, and 49.7% were classified as primary syphilis. Nontreponemal tests were reactive in 86.4% of cases, while treponemal tests were reactive in 56.8% of cases. Benzathine penicillin was prescribed to 75.3% of pregnant people. Sexual partners were treated in 41.2% of cases. Higher completion rates were observed in the notification forms for the variables “pregnancy trimester at diagnosis” (100.0%) and “nontreponemal test” (98.9%), which were classified as having “excellent” completeness. The lowest completion rates, classified as “poor,” were recorded for the variables “education level” (65.4%) and “clinical classification of syphilis” (70.6%) (Table 1).

**Table 1:** Absolute (n) and relative (%) frequencies, percentage (%), and degree of completeness of the clinical-epidemiological variables of syphilis during pregnancy cases. Regional Health Management Department of Ubá, 2018–2023 (n=837)

Variables	n (%)	Completeness (%)	Degree of completeness
**Race/skin color**		91.8	Good
White	237 (28.3)		
Black (Black and Brown)	518 (61.9)		
Asian	13 (1.6)		
Unknown	69 (8.2)		
**Education level**		65.4	Poor
Illiterate	1 (0.1)		
Incomplete elementary education	153 (18.3)		
Complete elementary education	209 (25.0)		
Complete high school	170 (20.3)		
Complete higher education	14 (1.7)		
Unknown	290 (34.6)		
**Gestational period of diagnosis** (trimesters)		100.0	Excellent
1st	225 (26.9)		
2nd	169 (20.2)		
3rd	302 (36.1)		
Gestational age unknown	141 (16.8)		
**Clinical classification of syphilis**		70.6	Poor
Primary	416 (49.7)		
Secondary	47 (5.6)		
Tertiary	26 (3.1)		
Latent	102 (12.2)		
Unknown	246 (29.4)		
**Nontreponemal test**		98.9	Excellent
Reactive	723 (86.4)		
Nonreactive	50 (6.0)		
Not performed	55 (6.6)		
Unknown	9 (1.0)		
**Treponemal test**		88.9	Fair
Reactive	475 (56.8)		
Nonreactive	50 (6.0)		
Not performed	219 (26.2)		
Unknown	93 (11.0)		
**Treatment prescribed to pregnant people**		94.1	Good
Benzathine penicillin	630 (75.3)		
Another scheme	52 (6.2)		
Not performed	106 (12.7)		
Unknown	49 (5.8)		
**Sexual partner treated**		89.1	Fair
Yes	345 (41.2)		
No	401 (47.9)		
Unknown	91 (10.9)		

The treatment prescribed to pregnant women with syphilis was classified as adequate for the clinical stage of the disease in 61.2% of cases, according to the PCDT of the MS. Meanwhile, 38.8% of prescriptions did not follow MS recommendations and were classified as inadequate. In the bivariate analysis, inadequate prescription was more prevalent: in the third pregnancy trimester (46.7%) compared to the first trimester (23.6%; p-value<0.001); among pregnant women with a nonreactive nontreponemal test (84.0%) compared to reactive test results (36.5%; p-value<0.001); when the treponemal test was not performed (39.7%) compared to reactive results (29.3%; p-value 0.006); and when the sexual partner was not treated (43.6%) compared to partners treated concurrently with the pregnant people (23.5%; p-value<0.001) (Table 2).

**Table 2:** Absolute (n) and relative (%) frequencies of the adequacy of prescribed treatment for gestational syphilis, according to clinical-epidemiological variables. Regional Health Management Department of Ubá, 2018–2023 (n=837)

Variables	Adequate prescription		p-value
	Yes n (%)	No n (%)	
**Race/skin color**			
White	139 (58.6)	98 (41.4)	Reference
Black (Black and Brown)	324 (62.5)	194 (37.5)	0.307
Asian	10 (76.9)	3 (23.1)	0.191
**Education level**			
Illiterate	1 (100.0)	-	1.000
Incomplete elementary education	109 (71.2)	44 (28.8)	0.140
Complete elementary education	148 (70.8)	61 (29.2)	0.132
Complete high school	108 (63.5)	62 (36.5)	Reference
Complete higher education	10 (71.4)	4 (28.6)	0.554
**Gestational period of diagnosis** (trimesters)			
1st	172 (76.4)	53 (23.6)	Reference
2nd	133 (78.7)	36 (21.3)	0.596
3rd	161 (53.3)	141 (46.7)	<0.001
**Nontreponemal test**			
Reactive	459 (63.5)	264 (36.5)	Reference
Nonreactive	8 (16.0)	42 (84.0)	<0.001
Not performed	39 (70.9)	16 (29.1)	0.269
**Treponemal test**			
Reactive	336 (70.7)	139 (29.3)	Reference
Nonreactive	29 (58.0)	21 (42.0)	0.063
Not performed	132 (60.3)	87 (39.7)	0.006
**Sexual partner treated**			
Yes	264 (76.5)	81 (23.5)	Reference
No	226 (56.4)	175 (43.6)	<0.001

In the multivariate analysis, inadequate prescription was more common among pregnant people diagnosed in the third trimester than among those diagnosed in the first trimester (OR 2.00; 95%CI 1.30;3.07; p-value 0.002). Pregnant people with a nonreactive nontreponemal test (OR 13.48; 95%CI 5.93; 30.64; p-value<0.001) and those who did not undergo the treponemal test (OR 1.65; 95%CI 1.13; 2.40; p-value 0.009) had higher odds of inadequate prescription. There were more prescription failures among pregnant women whose sexual partners were not treated (OR 1.91; 95%CI 1.34; 2.72; p-value<0.001) (Table 3).

**Table 3:** *Odds ratio* (OR) and 95% confidence interval (95%CI) for factors associated with the adequacy of the prescribed treatment for gestational syphilis. Regional Health Management Department of Ubá, 2018–2023 (n=837)

Variables	OR (95%CI)	p-value
**Gestational period of diagnosis** (trimesters)		
1st	Reference	<0.001
2nd	0.77 (0.45; 1.31)	0.334
3rd	2.00 (1.30; 3.07)	0.002
**Nontreponemal test**		
Reactive	Reference	<0.001
Nonreactive	13.48 (5.93; 30.64)	<0.001
Not performed	1.45 (0.74; 2.83)	0.274
**Treponemal test**		
Reactive	Reference	<0.001
Nonreactive	1.38 (0.69; 2.76)	0.368
Not performed	1.65 (1.13; 2.40)	0.009
**Sexual partner treated**		
Yes	Reference	<0.001
No	1.91 (1.34; 2.72)	<0.001

## Discussion 

This study evidenced a high occurrence of syphilis during pregnancy in the region of Ubá, Minas Gerais, and revealed significant deficiencies in the delivery of health care and epidemiological surveillance. Analysis of notification data showed variation in the completeness of variables. An association was identified between inadequate treatment prescription for pregnant women and other vulnerabilities in health services, related to delayed diagnosis, failures in conducting diagnostic tests, and the absence of treatment for sexual partners.

The study had inherent limitations due to the use of secondary data, which are subject to underreporting, incompleteness, and inconsistency. The low quality of completion for critical variables may have compromised the identification of epidemiological patterns. The assessment of treatment adequacy may have been affected, and there may have been possible misclassification of cases as primary syphilis. Nevertheless, the findings were relevant for highlighting weaknesses and guiding priority strategies for the region. The study provided useful evidence to support interventions against syphilis, generate hypotheses, and identify gaps for future research.

The increase in syphilis during pregnancy may be related to expanded testing, facilitated by the availability of rapid tests in primary health care (7) and by the implementation of the Syphilis Response Plan (“*Plano de Enfrentamento à Sífilis*”) in Minas Gerais, starting in 2021 (8). This state initiative provided financial incentives to municipalities to strengthen prevention and control actions, particularly regarding the early identification of infected pregnant people, intending to reduce the incidence of congenital syphilis (8). 

The scenario also signals the need to reinforce preventive actions, such as promoting condom use. It reflects the influence of multiple determinants, including a lack of awareness of the disease, access barriers, and individual vulnerabilities (4,5), underscoring the importance of an intersectoral, integrated approach.

The sociodemographic profile of pregnant people reflects historical structural inequalities and emphasizes the importance of equity in access to and quality of prenatal care, corroborating local, state, and national studies conducted in Brazil (6,15-18). A cohort based on more than 15 million births revealed that 87% of syphilis during pregnancy and 89% of congenital syphilis could have been prevented in Brazil if all women experienced the same risk as White women with more than 12 years of education (17), highlighting the impact of racial and social inequities on these outcomes. 

In Minas Gerais, it was demonstrated that between 2018 and 2019, higher education reduced the risk of syphilis during pregnancy by 75% (19). This study, in turn, showed that the higher frequency of infection among young, Black, and pregnant women with lower education levels reinforces this pattern and suggests that isolated measures of diagnosis and treatment have a limited impact when structural inequalities are not reduced.

Failures in diagnostic procedures were identified as a critical aspect. Predominant notifications in the third trimester raised the hypothesis of late diagnosis, which is known to be associated with a higher occurrence of congenital syphilis (20). However, the scenario in which the infection occurs in the final gestational period must also be considered, underscoring the importance of preventive and educational measures during prenatal care.

The predominance of primary syphilis cases suggested difficulties among health professionals in correctly identifying the clinical stage of infection, considering that primary syphilis diagnosis in pregnant women is uncommon (6,15). The high percentage of unclassified cases strengthens this suspicion.

The nonperformance of the treponemal test, despite its indication as an initial examination due to higher sensitivity (1), highlights weaknesses in diagnostic procedures. Although the case definition criteria allow notification of syphilis during pregnancy with only one reactive test, diagnostic confirmation should combine treponemal and nontreponemal tests (4). The rapid treponemal test is available in primary health care services in all 31 municipalities of the regional area, but its use is limited in some services (8). It should be noted that rapid testing is a cost-effective intervention that facilitates early diagnosis (1,5) and should be promoted.

Benzathine penicillin was the treatment prescribed for most pregnant people, but inadequate dosages and untreated cases were identified. Underprescription of penicillin for the treatment of latent, tertiary, and indeterminate syphilis cases was reported in Fortaleza between 2008 and 2010 (15). These findings reinforce the longstanding challenge of ensuring the correct clinical management of syphilis across different Brazilian settings, documented since 2015 (16,21,22).

This study identified an association between inadequate prescription and the absence of a treponemal test, suggesting that therapeutic decisions were often based solely on the titer of a nontreponemal test. Given the high prevalence of syphilis in Brazil, it is common for health professionals to interpret low titers as remnants of a past infection that does not require treatment. An increased likelihood of penicillin prescription has been reported among pregnant women with higher nontreponemal titers, suggesting that prescribers may rely on titer thresholds when making treatment decisions (23). Such practice is a frequent misconception, based on outdated interpretations from older literature that attributed a titer cut-off as an indicator of active infection (1).

A nonreactive nontreponemal test result was also associated with a higher chance of prescription errors. It is believed that interpreting a nonreactive result as the absence of active infection may have contributed to underprescription or failure to indicate treatment.

It is important to note that these tests are less sensitive in the early and latent stages of infection, potentially leading to underdiagnosis (24). These findings highlight important gaps in the implementation of diagnostic flowcharts for syphilis and in adherence to current protocols by health teams, underscoring the need for continuing education programs.

Late diagnosis during pregnancy and the absence of partner treatment were also associated with inadequate prescriptions, consistent with previous studies (25). Evidence indicates that pregnant people diagnosed in the third trimester had lower odds of receiving penicillin treatment in Brazil between 2010 and 2018 (23), reinforcing the hypothesis of shortcomings in clinical management combined with socioeconomic and organizational barriers within health services. Although these results support the importance of strengthening team training, interpretation should remain cautious and multifactorial, particularly given the scarcity of studies focused specifically on determinants of inadequate treatment for syphilis during pregnancy (25).

Partner treatment emerged as another critical issue, recorded in fewer than half of cases. Weaknesses in antenatal care practices and obstacles related to men’s access to health care contribute to low coverage of this intervention (26). The removal of partner treatment from the case definition criteria for congenital syphilis in 2017 may also have reduced the identification and follow-up of sexual partners (6,16). Overcoming this scenario is essential for disease control, given the risk of reinfection among pregnant women whose partners are not treated.

Although the notification form for syphilis during pregnancy allows the recording of the prescribed treatment, it does not capture information on whether the therapeutic regimen was actually initiated or completed. This gap underscores the need to consider additional challenges, including adherence and ongoing treatment. Primary health care services often lack adequate conditions for the management of syphilis, and some professionals refuse to administer penicillin, commonly citing insufficient infrastructure to manage potential anaphylaxis (9,22,27,28). However, the administration of benzathine penicillin in primary health care is well established as safe (29), and the persistence of this refusal represents an avoidable barrier to disease control.

Incomplete notifications are a persistent challenge for public health surveillance. Despite the observed variation, poor completeness was observed in key fields essential to case investigation and indicator calculation. This pattern is consistent with findings from records of syphilis in Bahia (2007–2017) (6) and in the city of Rio de Janeiro (2016–2020) (30). Contributing factors include the multiplicity of information systems, the large number of fields on reporting forms, the non-mandatory status of some items, and the heavy documentation burden in health services (30).

Such challenges hinder local epidemiological assessment, complicate comparisons across regions, and impair longitudinal monitoring, ultimately limiting the effectiveness of health policies. The quality of information is indispensable for public health planning and requires both awareness-raising and training of health professionals. Updating the notification forms and aligning them with SINAN are also urgent priorities (5).

The findings of this study revealed vulnerabilities within health services, highlighting the need to strengthen surveillance and the care provided to pregnant people. Recommended actions include strengthening service infrastructure, improving workforce training, and promoting integrated and shared care. Health professionals must be prepared to implement preventive actions, correctly interpret diagnostic tests, and apply current clinical guidelines. Epidemiological surveillance services should adopt routine monitoring of SINAN and promote continuous improvement in data quality. Strengthening coordination between surveillance, primary health care, and pharmaceutical services is strategic for reducing prescription errors and increasing the effectiveness of interventions. Updating data collection instruments—which currently lack information on treatment adherence in syphilis during pregnancy—could further strengthen surveillance and support timely intervention.

Improving clinical care and surveillance practices is essential to controlling syphilis during pregnancy and eliminating congenital syphilis through coordinated actions that reduce inequities, enhance the quality of care, and reinforce health information systems. Although this study reflects a specific regional context, its findings may help inform strategies in areas with similar organizational and sociodemographic profiles. Given the relevance of the results, future research should investigate determinants of inadequate treatment and support the design of effective interventions. 

## Data Availability

The database and analysis codes used in the study are available upon request from the corresponding author.
