# Supplementary material for: Epidemiological profile, completeness of notifications, and factors associated with the adequacy of treatment for syphilis during pregnancy: a cross-sectional study, Ubá, 2018–2023
Source: Epidemiol Serv Saude. 2026 Mar 20;35:e20250056. doi: 10.1590/S2237-96222026v35e20250056.en (PMC13008299; doi:10.1590/S2237-96222026v35e20250056.en)
Supplement: Translation

# Translation: Perfil epidemiológico, completude das notificações e fatores associados à adequação do tratamento da sífilis gestacional: estudo transversal, Ubá, 2018-2023

**DOI:** 10.1590/S2237-96222026v35e20250056.pt

**Authors:** Priscila Teixeira Silva, Luana Vieira Toledo, Brunnella Alcantara Chagas de Freitas, Valker Araújo Feitosa

**Affiliations:** 1 Universidade Federal de Viçosa, Departamento de Medicina e Enfermagem, Viçosa, MG, Brasil; 2 Universidade de São Paulo, Faculdade de Ciências Farmacêuticas, São Paulo, SP, Brasil

**Keywords:** Sífilis, Gestantes, Infecções Sexualmente Transmissíveis, Notificação de Doenças, Estudos Transversais

## Abstract

**Objetivo:**

Analisar o perfil epidemiológico da sífilis gestacional, a completude das notificações e os fatores associados à adequação do tratamento em Ubá, região de Minas Gerais, entre 2018 e 2023.

**Métodos:**

Estudo transversal com registros do Sistema de Informação de Agravos de Notificação. Calcularam-se a taxa de detecção anual de sífilis gestacional e a regressão logística para avaliar o tratamento. A completude dos dados foi calculada e categorizada.

**Resultados:**

A taxa de detecção variou de 17,7 casos por 1 mil nascidos vivos em 2019 a 41,3 em 2022. As gestantes apresentaram mediana de 23 anos (intervalo interquartil 19; 28), sendo 61,9% de raça/cor da pele negra e 36,1% diagnosticadas no terceiro trimestre. A penicilina foi indicada em 75,3% dos casos, e o tratamento da parceria ocorreu em 41,2%. A completude foi excelente para as variáveis “período gestacional do diagnóstico” (100,0%) e “teste não treponêmico” (98,9%); ruim para “escolaridade” (65,4%) e “classificação clínica” (70,6%). A prescrição inadequada foi maior para gestantes diagnosticadas no terceiro trimestre em comparação ao diagnóstico no primeiro trimestre (*odds ratio* [OR] 2,00; intervalo de confiança de 95% [IC95%] 1,30; 3,07). As gestantes com resultado não reagente para o teste não treponêmico (OR 13,48; IC95% 5,93; 30,64) e que não realizaram o teste treponêmico (OR 1,65; IC95% 1,13; 2,40) apresentaram maiores chances de prescrição inadequada. Houve mais falhas de prescrição para as gestantes cujas parcerias sexuais não foram tratadas (OR 1,91; IC95% 1,34; 2,72).

**Conclusão:**

Observaram-se elevada taxa de detecção e falhas na completude das notificações e no tratamento prescrito das gestantes com sífilis.

**Aspectos éticos**

Esta pesquisa respeitou os princípios éticos, obtendo os seguintes dados de aprovação:

Comitê de ética em pesquisa: Universidade Federal de Viçosa

Número do parecer: 6.312.070

Data de aprovação: 20/9/2023

Certificado de apresentação de apreciação ética: 73354323.9.0000.5153

Registro do consentimento livre e esclarecido: Dispensado.

## Introdução 

A sífilis é uma doença secular que persiste como desafio de saúde pública. Uma das principais preocupações para o controle dessa infecção sexualmente transmissível é o acometimento da gestante, que pode transmiti-la para o concepto, ocasionando a sífilis congênita. A transmissão vertical é evitável a partir do diagnóstico precoce e do tratamento adequado da gestante durante o pré-natal (1).

Apesar de amplo conhecimento sobre estratégias preventivas, de disponibilidade de testes sensíveis e de tratamento efetivo de baixo custo, a sífilis é a segunda principal causa de natimortos evitáveis no mundo. A estimativa global em 2022 foi de 700 mil casos de sífilis congênita e mais de 200 mil óbitos fetais e neonatais (2).

Em 2007, a Organização Mundial da Saúde lançou uma iniciativa para eliminar a transmissão vertical, a fim de aumentar o acesso à testagem e ao tratamento para gestantes. Estabeleceu-se um processo de validação oficial para que os países fossem reconhecidos ao atingirem esse objetivo e considerou-se aceitável a incidência de até 0,5 caso de sífilis congênita por 1 mil nascidos vivos (3). 

O Brasil tem se confrontado com números distantes das metas para eliminação. Em 2023, apresentou incidência de 34 casos de sífilis gestacional e aproximadamente 10 casos de sífilis congênita por 1 mil nascidos vivos (4).

Os dados nacionais sobre sífilis são provenientes do Sistema de Informação de Agravos de Notificação (Sinan). A notificação compulsória se dá através do preenchimento de formulários padronizados pelo profissional de saúde que identificou o caso, com posterior envio ao serviço de vigilância epidemiológica para inserção dos dados no Sinan. 

A vigilância da sífilis é uma importante ferramenta de acompanhamento, que permite monitorar o comportamento da doença, além de fundamentar o planejamento de medidas de prevenção e de controle (5). A qualidade dos registros, a partir da completude e da consistência de todos os dados informados, é essencial para que os indicadores de saúde cumpram suas finalidades (6).

Minas Gerais tem notificado um aumento gradual da sífilis e o tratamento inadequado das gestantes se mostra importante desafio a ser superado. Ressalta-se a fragilidade na completude das notificações e as implicações para o planejamento de ações prioritárias, o que evidencia a necessidade de qualificar o banco de dados (7). Em 2023, o estado registrou mais de 30 casos de sífilis gestacional e aproximadamente 10 casos de sífilis congênita por 1 mil nascidos vivos (7). Desde 2021, a regional de Ubá apresentou taxas superiores às estaduais e nacionais (4,7,8).

Caracterizar os casos e identificar fatores relacionados à qualidade dos cuidados prestados em uma região faz-se fundamental para a formulação de políticas de saúde efetivas (9). O diagnóstico do cenário regional aponta as reais necessidades da população em questão e permite propor ações que considerem suas particularidades, a fim de avançar na prevenção e no controle da sífilis.

Este estudo objetivou analisar o perfil epidemiológico da sífilis gestacional, a completude das notificações e os fatores associados à adequação do tratamento em Ubá, região de Minas Gerais, entre 2018 e 2023.

## Métodos 

### Delineamento 

Trata-se de estudo transversal, de caráter descritivo-analítico, a partir de notificações de sífilis gestacional registradas no Sinan, em residentes da regional de Ubá, Minas Gerais.

### Contexto 

Os 583 municípios mineiros estão organizados em 14 macrorregiões e 89 microrregiões de saúde, com o objetivo de ordenar a oferta de serviços em suas respectivas áreas de abrangência. 

A regional de Ubá localiza-se na Zona da Mata e compõe a macrorregião Sudeste. Essa regional engloba a: (i) microrregião de Muriaé, composta por 11 municípios, com população estimada em 173.744 habitantes em 2020; e (ii) microrregião de Ubá, constituída de 20 municípios e população estimada em 314.647 habitantes em 2020 (10). 

Sua definição como cenário deste estudo justificou-se pela relevância epidemiológica do território, que vem se destacando com altas incidências de sífilis, com superação das taxas estaduais e nacionais (4,7,8).

### Participantes 

Compuseram este estudo os casos de sífilis gestacional notificados entre residentes dos 31 municípios que compõem a regional, com diagnóstico entre 2018 e 2023. O recorte temporal referiu-se à necessidade de padronizar os critérios de definição de casos, atualizados em 2017. Considerou-se caso de sífilis gestacional a mulher que, durante o pré-natal, o parto e o puerpério, apresentou teste treponêmico e não treponêmico reagente(s), independentemente de sintomatologia (11).

Excluíram-se do estudo notificações automaticamente classificadas pelo sistema como “descartado”, por não atenderem aos critérios de definição.

### Variáveis 

A notificação de sífilis gestacional foi preenchida pelos profissionais dos serviços de saúde, mediante formulário padronizado. O estudo considerou as seguintes variáveis da ficha: idade; raça/cor da pele (branca, preta, amarela, parda, indígena); escolaridade (analfabeto, primeira à quarta série incompleta do ensino fundamental, quarta série completa do ensino fundamental, quinta à oitava série incompleta do ensino fundamental, ensino fundamental completo, ensino médio incompleto, ensino médio completo, educação superior incompleta, educação superior completa); período gestacional do diagnóstico (primeiro trimestre, segundo trimestre, terceiro trimestre, idade gestacional ignorada); classificação clínica da sífilis (primária, secundária, terciária, latente); teste não treponêmico (reagente, não reagente, não realizado); teste treponêmico (reagente, não reagente, não realizado); tratamento prescrito à gestante (penicilina benzatina 2,4 milhões unidades internacionais, penicilina benzatina 4,8 milhões unidades internacionais, penicilina benzatina 7,2 milhões unidades internacionais, outro esquema, não realizado); e parceria sexual tratada (sim, não).

A seleção dessas variáveis deu-se pelo fato de o preenchimento ser obrigatório ou essencial, fundamentais para vigilância dos casos e cálculo dos indicadores epidemiológicos e operacionais.

A partir das variáveis “classificação clínica da sífilis” e “tratamento prescrito à gestante”, os pesquisadores avaliaram se a prescrição foi adequada para a fase clínica da infecção, com a consideração dos Protocolos Clínicos e Diretrizes Terapêuticas do Ministério da Saúde (MS). A avaliação se deu de forma manual, com dupla checagem. Consideraram-se adequadas: (i) uma, duas ou três doses de penicilina benzatina 2,4 unidades internacionais em caso de sífilis primária e secundária; e (ii) três doses de penicilina benzatina 2,4 milhões unidades internacionais em caso de sífilis latente, terciária e quando a variável “classificação clínica da sífilis” estava ignorada/em branco (12). Com base nessa classificação, criou-se a variável “prescrição de tratamento adequado”, com categorias “sim” e “não”. 

Além das prescrições inadequadas, incluíram-se, na categoria “não”, os casos cujo campo “tratamento prescrito à gestante” estava assinalado com a opção “não realizado”. Essa decisão seguiu o indicador adotado pelo MS para monitorar a cobertura de gestantes tratadas adequadamente para sífilis, que considerou ambas as situações como inadequadas por não prevenirem a transmissão vertical (12).

### Fonte de dados e mensuração

Dados de notificação foram coletados da base nominal do Sinan da regional em 1^o^ de março de 2024. Dados sobre nascidos vivos foram extraídos em 31 de outubro de 2024, do banco publicizado pelo Departamento de Informática do Sistema Único de Saúde, disponível no Portal da Vigilância em Saúde de Minas Gerais (13), aplicando-se filtros para o ano de nascimento e a microrregião de interesse.

Alguns ajustes foram realizados no banco extraído do Sinan, para melhor análise e avaliação dos resultados.

- Houve identificação e exclusão das duplicidades, quando o caso foi notificado mais de uma vez durante a mesma gestação. Para identificação das duplicidades, consideraram-se: nome, nome da mãe, data de nascimento e data da notificação. No caso de duplicidade, manteve-se apenas o primeiro registro a dar entrada na base de dados.
- Agruparam-se as categorias “preta” e “parda” como “negra” na variável “raça/cor da pele”, conforme quesito utilizado pelo Instituto Brasileiro de Geografia e Estatística.
- Agrupou-se a variável “escolaridade” em: analfabeto, ensino fundamental incompleto, ensino fundamental completo, ensino médio completo e ensino superior completo.
- Agrupou-se a variável “tratamento prescrito à gestante” em: penicilina benzatina, outro esquema e não realizado.

### Métodos estatísticos

Calculou-se a taxa de detecção anual de sífilis em gestantes a partir da utilização do número absoluto de casos como numerador e do número de nascidos vivos como denominador, multiplicado por 1 mil.

As variáveis de interesse para caracterização do perfil clínico-epidemiológico foram descritas por meio de frequências simples e relativas, mediana e intervalo interquartil. Na estatística descritiva, os “dados faltantes” foram incluídos na categoria “ignorado”. Para avaliação de completude, calculou-se o percentual de preenchimento de cada variável. Campos com ausência de informação e preenchidos com categoria “ignorado” foram considerados incompletos. A completude foi categorizada conforme a seguinte classificação: excelente (completude>95%), bom (completude entre 90%-95%), regular (completude entre 80%-90%), ruim (completude entre 50%-80%) e muito ruim (completude <50%) (14).

Considerou-se como variável dependente a “prescrição de tratamento adequado”. Na análise bivariada, avaliou-se a associação de cada variável independente através dos testes qui-quadrado de Pearson. Quando as tabelas de contingência apresentaram frequências esperadas menores que cinco, utilizou-se o exato de Fisher. Dados faltantes e ignorados das variáveis independentes foram desconsiderados nessa etapa. 

Na análise multivariada, empregou-se regressão logística para estimar a magnitude da associação entre as variáveis independentes e a prescrição de tratamento adequado (variável dependente). Calcularam-se a razão de chances (*odds ratio*, OR) e os intervalos de confiança de 95% (IC95%). O modelo foi considerado bem ajustado pelo teste de Hosmer-Lemeshow (p-valor 0,657). A significância estatística foi avaliada pelo teste de Wald, com p-valor<0,05. Todas as análises foram realizadas no programa IBM SPSS Statistics.

## Resultados 

A base de dados original apresentava 853 registros. Excluíram-se da análise 4 casos classificados como “descartados” e 12 duplicidades. Entre 2018 e 2023, identificaram-se 837 casos de sífilis gestacional e, em 2022, houve a maior taxa de detecção, 41,3 casos por 1 mil nascidos vivos (Figura 1).

**Figura 1:** Taxa de detecção de sífilis em gestante (casos por 1 mil nascidos vivos), segundo ano de diagnóstico. Regional de Ubá, 2018-2023 (n=837)

As gestantes com sífilis apresentaram mediana de idade de 23 anos (intervalo interquartil 19; 28) e 61,9% eram negras. Observou-se que 36,1% dos diagnósticos ocorreram no terceiro trimestre gestacional e 49,7% foram classificados como sífilis primária. Os testes não treponêmicos apresentaram resultado reagente em 86,4% dos casos, enquanto os treponêmicos foram reagentes em 56,8% dos casos. A penicilina benzatina foi indicada para 75,3% das gestantes. As parcerias sexuais foram tratadas em 41,2% dos casos. Observou-se maior percentual de preenchimento nas fichas de notificação para as variáveis “período gestacional do diagnóstico” (100,0%) e “teste não treponêmico” (98,9%), que apresentaram completude “excelente”. Os menores percentuais de preenchimento apresentaram completude “ruim” e foram registrados para as variáveis “escolaridade” (65,4%) e “classificação clínica da sífilis” (70,6%) (Tabela 1).

**Tabela 1:** Frequências absoluta (n) e relativa (%), percentual (%) e grau de completude das variáveis clínico-epidemiológicas dos casos de sífilis gestacional. Regional de Ubá, 2018-2023 (n=837)

Variáveis	n (%)	Completude (%)	Grau de completude
**Raça/cor da pele**		91,8	Bom
Branca	237 (28,3)		
Negra (preta e parda)	518 (61,9)		
Amarela	13 (1,6)		
Ignorado	69 (8,2)		
Escolaridade		65,4	Ruim
Analfabeto	1 (0,1)		
Ensino fundamental incompleto	153 (18,3)		
Ensino fundamental completo	209 (25,0)		
Ensino médio completo	170 (20,3)		
Ensino superior completo	14 (1,7)		
Ignorado	290 (34,6)		
**Período gestacional do diagnóstico** (trimestres)		100,0	Excelente
1°	225 (26,9)		
2°	169 (20,2)		
3°	302 (36,1)		
Idade gestacional ignorada	141 (16,8)		
**Classificação clínica da sífilis**		70,6	Ruim
Primária	416 (49,7)		
Secundária	47 (5,6)		
Terciária	26 (3,1)		
Latente	102 (12,2)		
Ignorado	246 (29,4)		
**Teste não treponêmico**		98,9	Excelente
Reagente	723 (86,4)		
Não reagente	50 (6,0)		
Não realizado	55 (6,6)		
Ignorado	9 (1,0)		
**Teste treponêmico**		88,9	Regular
Reagente	475 (56,8)		
Não reagente	50 (6,0)		
Não realizado	219 (26,2)		
Ignorado	93 (11,0)		
**Tratamento prescrito à gestante**		94,1	Bom
Penicilina benzatina	630 (75,3)		
Outro esquema	52 (6,2)		
Não realizado	106 (12,7)		
Ignorado	49 (5,8)		
**Parceria sexual tratada**		89,1	Regular
Sim	345 (41,2)		
Não	401 (47,9)		
Ignorado	91 (10,9)		

A prescrição oferecida à gestante com sífilis foi classificada como adequada para a fase clínica da doença em 61,2% dos casos, com a consideração dos Protocolos Clínicos e Diretrizes Terapêuticas do MS. Já 38,8% das prescrições realizadas não seguiram as recomendações do MS e receberam classificação de inadequadas.

Na análise bivariada, a prescrição inadequada foi mais prevalente: no terceiro trimestre gestacional (46,7%) em comparação ao primeiro trimestre (23,6%; p-valor<0,001); entre gestantes com teste não treponêmico não reagente (84,0%) em comparação ao teste reagente (36,5%; p-valor<0,001); quando não realizado o teste treponêmico (39,7%) em comparação ao teste reagente (29,3%; p-valor 0,006); e na ausência do tratamento da parceria sexual (43,6%) em comparação às parcerias tratadas concomitantemente às gestantes (23,5%; p-valor<0,001) (Tabela 2).

**Tabela 2:** Frequências absoluta (n) e relativa (%) da adequação da prescrição terapêutica para sífilis gestacional, segundo variáveis clínico-epidemiológicas. Regional de Ubá, 2018-2023 (n=837)

Variáveis	Prescrição adequada		p-valor
	Sim n (%)	Não n (%)	
**Raça/cor da pele**			
Branca	139 (58,6)	98 (41,4)	Referência
Negra (preta e parda)	324 (62,5)	194 (37,5)	0,307
Amarela	10 (76,9)	3 (23,1)	0,191
**Escolaridade**			
Analfabeto	1 (100,0)	-	1,000
Ensino fundamental incompleto	109 (71,2)	44 (28,8)	0,140
Ensino fundamental completo	148 (70,8)	61 (29,2)	0,132
Ensino médio completo	108 (63,5)	62 (36,5)	Referência
Ensino superior completo	10 (71,4)	4 (28,6)	0,554
**Período gestacional do diagnóstico** (trimestres)			
1°	172 (76,4)	53 (23,6)	Referência
2°	133 (78,7)	36 (21,3)	0,596
3°	161 (53,3)	141 (46,7)	<0,001
**Teste não treponêmico**			
Reagente	459 (63,5)	264 (36,5)	Referência
Não reagente	8 (16,0)	42 (84,0)	<0,001
Não realizado	39 (70,9)	16 (29,1)	0,269
**Teste treponêmico**			
Reagente	336 (70,7)	139 (29,3)	Referência
Não reagente	29 (58,0)	21 (42,0)	0,063
Não realizado	132 (60,3)	87 (39,7)	0,006
**Parceria sexual tratada**			
Sim	264 (76,5)	81 (23,5)	Referência
Não	226 (56,4)	175 (43,6)	<0,001

Na análise multivariada, a prescrição inadequada foi maior para gestantes diagnosticadas no terceiro trimestre em comparação ao diagnóstico no primeiro trimestre (OR 2,00; IC95% 1,30; 3,07; p-valor 0,002). As gestantes com resultado não reagente para o teste não treponêmico (OR 13,48; IC95% 5,93; 30,64; p-valor<0,001) e que não realizaram o teste treponêmico (OR 1,65; IC95% 1,13; 2,40; p-valor 0,009) apresentaram maior chances de prescrição inadequada. Houve mais falhas de prescrição para as gestantes cujas parcerias sexuais não foram tratadas (OR 1,91; IC95% 1,34; 2,72; p-valor<0,001) (Tabela 3).

**Tabela 3:** Razão de chances (*odds ratio*, OR) e intervalo de confiança de 95% (IC95%) dos fatores associados à adequação da prescrição terapêutica para sífilis gestacional. Regional de Ubá, 2018-2023 (n=837)

Variáveis	OR (IC95%)	p-valor
**Período gestacional do diagnóstico** (trimestres)		
1°	Referência	<0,001
2°	0,77 (0,45; 1,31)	0,334
3°	2,00 (1,30; 3,07)	0,002
**Teste não treponêmico**		
Reagente	Referência	<0,001
Não reagente	13,48 (5,93; 30,64)	<0,001
Não realizado	1,45 (0,74; 2,83)	0,274
**Teste treponêmico**		
Reagente	Referência	<0,001
Não reagente	1,38 (0,69; 2,76)	0,368
Não realizado	1,65 (1,13; 2,40)	0,009
**Parceria sexual tratada**		
Sim	Referência	<0,001
Não	1,91 (1,34; 2,72)	<0,001

## Discussão 

Este estudo evidenciou elevada ocorrência de sífilis gestacional na região de Ubá, Minas Gerais, e revelou importantes deficiências na assistência à saúde e na vigilância epidemiológica. A análise dos dados de notificação demonstrou variação na completude das variáveis. Identificou-se associação da prescrição inadequada para tratamento das gestantes com outras vulnerabilidades dos serviços de saúde, relacionadas a diagnóstico inoportuno, falhas na condução dos testes diagnósticos e ausência de tratamento das parcerias sexuais.

O estudo teve limitações inerentes ao uso de dados secundários, sujeitos à subnotificação, incompletude e inconsistência. A baixa qualidade do preenchimento de variáveis críticas pode ter comprometido a identificação de padrões epidemiológicos. A avaliação da adequação das prescrições pode ter sido afetada e pode ter havido possível classificação equivocada de casos como sífilis primária. Mesmo assim, os achados foram relevantes para apontar fragilidades e nortear estratégias prioritárias para a região. O estudo produziu evidências úteis para subsidiar intervenções de enfrentamento à sífilis, levantar hipóteses e apontar lacunas para que futuras pesquisas aprofundem a investigação.

O crescimento da sífilis gestacional pode estar relacionado à ampliação da testagem, favorecida pela disponibilidade de testes rápidos na atenção básica à saúde (7) e pela implementação do Plano de Enfretamento à Sífilis em Minas Gerais, a partir de 2021 (8). Essa iniciativa estadual destinou incentivo financeiro aos municípios para fortalecer as ações de prevenção e de controle, especialmente no que se diz respeito à identificação precoce das gestantes infectadas, com o objetivo de reduzir a incidência de sífilis congênita (8). 

O cenário identificado também sinaliza a necessidade de reforçar as ações de prevenção, como o incentivo ao uso de preservativos sexuais, e remete à influência de múltiplos determinantes, como falta de conscientização sobre a doença, barreiras de acesso e vulnerabilidades individuais (4,5), o que reforça a importância da abordagem intersetorial e integrada.

O perfil sociodemográfico das gestantes reflete desigualdades estruturais históricas e enfatiza a importância da equidade no acesso e na assistência pré-natal, o que corrobora estudos locais, estaduais e nacionais realizados no Brasil (6,15-18). Uma coorte baseada em mais de 15 milhões de nascimentos revelou que 87% da sífilis gestacional e 89% da sífilis congênita teriam sido evitadas no Brasil se todas as mulheres experimentassem o mesmo risco que as de raça/cor da pele branca com mais de 12 anos de estudo (17), o que evidencia o impacto das iniquidades raciais e sociais nesses desfechos. 

Em Minas Gerais, foi demonstrado que, entre 2018 e 2019, a maior escolaridade reduziu em 75% a chance de sífilis gestacional (19). Este estudo, por sua vez, mostrou que a maior frequência da infecção entre gestantes jovens, negras e com baixa escolaridade reforça esse padrão e sugere que medidas isoladas de diagnóstico e de tratamento, sem redução das desigualdades estruturais, têm impacto limitado.

As falhas na condução do diagnóstico foram um aspecto crítico identificado. A notificação predominante no terceiro trimestre levantou a hipótese de diagnóstico tardio, sabidamente associado à maior ocorrência de sífilis congênita (20). Porém, deve-se considerar o cenário em que a infecção ocorre no último período gestacional, o que reforça a relevância das ações preventivas e educativas durante o pré-natal.

A predominância dos casos de sífilis primária sugeriu dificuldades dos profissionais em identificar corretamente a fase clínica da infecção, com a consideração de que o diagnóstico de sífilis primária na gestante é incomum (6,15). O elevado percentual de casos não classificados fortalece essa suspeita.

A não realização do teste treponêmico, apesar de sua indicação como exame inicial, devido à maior sensibilidade (1), reforça a fragilidade na condução do diagnóstico. Embora os critérios de definição permitam a notificação de sífilis gestacional com apenas um teste reagente, a confirmação diagnóstica deve combinar testes treponêmico e não treponêmico (4). O teste rápido treponêmico está disponível na atenção básica dos 31 municípios da regional, mas a utilização é escassa em alguns serviços (8). Destaca-se que a testagem rápida é uma intervenção custo-efetiva que favorece o diagnóstico precoce (1,5) e deve ser fomentada.

A penicilina benzatina foi o tratamento prescrito para a maioria das gestantes, mas identificaram-se posologias inadequadas e casos não tratados. Foi registrado subprescrição de penicilina para tratamento de casos de sífilis latente, terciária e indeterminada em Fortaleza, entre 2008 e 2010 (15). Esses resultados reforçam o desafio que é garantir o manejo clínico correto da sífilis em diferentes contextos brasileiros, desde 2015 (16,21,22).

Este estudo identificou associação da prescrição inadequada à ausência do teste treponêmico, o que sugeriu que a decisão terapêutica foi baseada na análise isolada do título de um teste não treponêmico. Diante da alta prevalência de sífilis no Brasil, é prática comum que os profissionais interpretem títulos mais baixos como remanescentes de uma infecção antiga que não precisa de tratamento. Foi identificado aumento nas chances de tratamento com penicilina em casos de sífilis materna com títulos elevados de testes não treponêmicos, o que sugeriu que os prescritores podem usar um limite de título no processo de decisão (23). Tal conduta é um equívoco frequente, decorrente de interpretações desatualizadas, baseadas em textos antigos, que atribuíam um ponto de corte no título como indicativo de infecção ativa (1).

O teste não treponêmico não reagente também esteve associado à maior chance de erros de prescrição. Acredita-se que a interpretação de um resultado não reagente como ausência de infecção ativa pode ter contribuído para a subprescrição ou a falta de indicação de tratamento.

Há de se considerar que tais testes são menos sensíveis nas fases inicial e latente da infecção, com risco potencial de subdiagnóstico (24). Esses achados evidenciam lacunas na condução dos fluxogramas para diagnóstico da sífilis e adesão das equipes de saúde aos protocolos vigentes, o que aponta para a importância dos programas de educação continuada.

O diagnóstico no final da gestação e a ausência de tratamento da parceria sexual também estiveram associados à prescrição inadequada, o que corrobora outros achados (25). Foi identificado que gestantes diagnosticadas no terceiro trimestre apresentaram menores chances de receber tratamento com penicilina no Brasil, entre 2010 e 2018 (23), o que reforça a hipótese de fragilidades no manejo clínico, somadas a dificuldades socioeconômicas e organizacionais dos serviços de saúde. Ainda que esses resultados sustentem a importância de capacitação das equipes, a interpretação deve ser cautelosa e multifatorial, especialmente diante da escassez de estudos específicos sobre fatores relacionados à inadequação terapêutica da sífilis gestacional (25).

O tratamento das parcerias sexuais das gestantes foi outro aspecto crítico identificado, registrado em menos da metade dos casos. Fragilidades na abordagem do pré-natal e dificuldades relacionadas à assistência à saúde do homem comprometem a cobertura dessa intervenção (26). A exclusão do tratamento da parceria materna dos critérios de definição de caso de sífilis congênita, em 2017, também pode ter contribuído para reduzir a captação e o acompanhamento das parcerias (6,16). Superar esse cenário é essencial para o controle da doença, com a consideração do risco de reinfecção em gestantes cujas parcerias não receberam tratamento.

Embora a ficha de notificação de sífilis gestacional permita registrar o tratamento prescrito, ela não contempla informações sobre a efetiva realização e conclusão do regime terapêutico. Essa lacuna reforça a necessidade de considerar desafios adicionais, como acesso, adesão e continuidade do tratamento. No Brasil, há predominância de serviços de atenção básica à saúde em condições inadequadas para o manejo da sífilis e recusa de alguns profissionais em administrar a penicilina, que se justificam com a ausência de infraestrutura para lidar com possíveis reações anafiláticas (9,22,27,28). Contudo, a aplicação da penicilina benzatina na atenção básica é comprovadamente segura (29), e a persistência dessa recusa constitui um obstáculo evitável para o controle da doença.

A incompletude das notificações é um desafio para vigilância em saúde. Apesar da variação observada, verificou-se qualidade ruim no preenchimento de campos fundamentais para investigação dos casos e cálculo de indicadores. Esse padrão foi semelhante a registros de sífilis na Bahia, entre 2007 e 2017 (6), e no município do Rio de Janeiro, entre 2016 e 2020 (30), o que pode estar relacionado à multiplicidade de sistemas, à quantidade de campos presentes nas fichas, à não obrigatoriedade do preenchimento de alguns itens e à sobrecarga de formulários nos serviços (30).

Essa realidade compromete a avaliação epidemiológica local, dificulta a comparação com outros territórios e prejudica o monitoramento temporal, o que pode limitar a efetividade das políticas de saúde. A qualidade da informação é indispensável para o planejamento em saúde pública, o que demanda sensibilização e capacitação dos profissionais. Também é urgente atualizar os formulários de notificação e alinhá-los ao Sinan (5).

Os resultados deste estudo apontaram vulnerabilidades nos serviços, o que evidenciou a necessidade de fortalecer a vigilância e a assistência à saúde das gestantes. Recomendam-se estruturação dos serviços, qualificação das equipes de saúde e fomento ao cuidado compartilhado e integral. Os profissionais devem estar aptos a promover ações preventivas, interpretar corretamente os testes diagnósticos e aplicar as diretrizes vigentes para manejo dos casos. Os serviços de vigilância epidemiológica devem adotar rotinas de monitoramento do Sinan e promover a melhoria contínua da qualidade dos dados. Acredita-se que a articulação entre vigilância, atenção básica à saúde e assistência farmacêutica é estratégica para reduzir os erros de prescrição e ampliar e a efetividade das intervenções. A atualização dos instrumentos de coleta de dados, que carecem de informação sobre adesão ao tratamento da sífilis gestacional, também poderia fortalecer a vigilância para intervenção em tempo oportuno.

Qualificar as práticas de atenção e de vigilância em saúde é fundamental para o controle da sífilis gestacional e a eliminação da sífilis congênita, por meio de ações articuladas para reduzir iniquidades, aprimorar o cuidado e fortalecer os sistemas de informação. Embora este estudo reflita a realidade regional, os resultados podem contribuir para formulação de estratégias em regiões com contextos organizacionais e sociodemográficos semelhantes. Dada a relevância dos achados, pesquisas futuras poderão investigar fatores determinantes da inadequação terapêutica e subsidiar intervenções eficazes. 

## Data Availability

O banco de dados e os códigos de análise utilizados na pesquisa estão disponíveis sob demanda à autora correspondente.
